# Biomarkers Associated With Severe COVID-19 Among Populations With High Cardiometabolic Risk: A 2-Sample Mendelian Randomization Study

**DOI:** 10.1001/jamanetworkopen.2023.25914

**Published:** 2023-07-27

**Authors:** Tushar Sood, Nicolas Perrot, Michael Chong, Pedrum Mohammadi-Shemirani, Maha Mushtaha, Darryl Leong, Sumathy Rangarajan, Sibylle Hess, Salim Yusuf, Hertzel C. Gerstein, Guillaume Paré, Marie Pigeyre

**Affiliations:** 1 Population Health Research Institute, Hamilton, Ontario, Canada; 2 Temerty Faculty of Medicine, University of Toronto, Toronto, Ontario, Canada; 3 Department of Pathology and Molecular Medicine, McMaster University, Hamilton, Ontario, Canada; 4 Thrombosis and Atherosclerosis Research Institute, Hamilton, Ontario, Canada; 5 Deep Genomics Inc, Toronto, Ontario, Canada; 6 Department of Medicine, McMaster University, Hamilton, Ontario, Canada; 7 Global Medical Diabetes, Sanofi, Frankfurt, Germany

## Abstract

**Importance:**

Cardiometabolic parameters are established risk factors for COVID-19 severity. The identification of causal or protective biomarkers for COVID-19 severity may facilitate the development of novel therapies.

**Objective:**

To identify protein biomarkers that promote or reduce COVID-19 severity and that mediate the association of cardiometabolic risk factors with COVID-19 severity.

**Design, Setting, and Participants:**

This genetic association study using 2-sample mendelian randomization (MR) was conducted in 2022 to investigate associations among cardiometabolic risk factors, circulating biomarkers, and COVID-19 hospitalization. Inputs for MR included genetic and proteomic data from 4147 participants with dysglycemia and cardiovascular risk factors collected through the Outcome Reduction With Initial Glargine Intervention (ORIGIN) trial. Genome-wide association study summary statistics were obtained from (1) 3 additional independent plasma proteome studies, (2) genetic consortia for selected cardiometabolic risk factors (including body mass index [BMI], type 2 diabetes, type 1 diabetes, and systolic blood pressure; all n >10 000), and (3) the COVID-19 Host Genetics Initiative (n = 5773 hospitalized and 15 497 nonhospitalized case participants with COVID-19). Data analysis was performed in July 2022.

**Exposures:**

Genetically determined concentrations of 235 circulating proteins assayed with a multiplex biomarker panel from the ORIGIN trial for the initial analysis.

**Main Outcomes and Measures:**

Hospitalization status of individuals from the COVID-19 Host Genetics Initiative with a positive COVID-19 test result.

**Results:**

Among 235 biomarkers tested in samples totaling 22 101 individuals, MR analysis showed that higher kidney injury molecule-1 (KIM-1) levels reduced the likelihood of COVID-19 hospitalization (odds ratio [OR] per SD increase in KIM-1 levels, 0.86 [95% CI, 0.79-0.93]). A meta-analysis validated the protective association with no observed directional pleiotropy (OR per SD increase in KIM-1 levels, 0.91 [95% CI, 0.88-0.95]). Of the cardiometabolic risk factors studied, only BMI was associated with KIM-1 levels (0.17 SD increase in biomarker level per 1 kg/m^2^ [95% CI, 0.08-0.26]) and COVID-19 hospitalization (OR per 1-SD biomarker level, 1.33 [95% CI, 1.18-1.50]). Multivariable MR analysis also revealed that KIM-1 partially mitigated the association of BMI with COVID-19 hospitalization, reducing it by 10 percentage points (OR adjusted for KIM-1 level per 1 kg/m^2^, 1.23 [95% CI, 1.06-1.43]).

**Conclusions and Relevance:**

In this genetic association study, KIM-1 was identified as a potential mitigator of COVID-19 severity, possibly attenuating the increased risk of COVID-19 hospitalization among individuals with high BMI. Further studies are required to better understand the underlying biological mechanisms.

## Key Points

**Question:**

What are the mediators linking cardiometabolic risk factors and severe COVID-19?

**Findings:**

In this genetic association study that used mendelian randomization to analyze 235 biomarkers in samples from 22 101 individuals, the biomarker kidney injury molecule-1 (KIM-1) was associated with reduced COVID-19 hospitalization and KIM-1 circulating levels were upregulated with increased body mass index (BMI).

**Meaning:**

These findings suggest that KIM-1 may attenuate the association of BMI with COVID-19 severity.

## Teaser

This genetic association study uses a 2-sample mendelian randomization approach to investigate associations among cardiometabolic risk factors, circulating biomarkers, and COVID-19 hospitalization.

## Introduction

Cardiometabolic parameters such as obesity,^1^ type 2 diabetes (T2D),^2^ and hypertension^3^ are established risk factors for complications associated with COVID-19. For instance, individuals with obesity have a 2.1-fold increase in hospitalization, a 1.7-fold increase in admission to the intensive care unit, and a 1.5-fold increase in death as a result of severe COVID-19.^4^ Similar odds were also reported for T2D and hypertension.^4^

Although epidemiologic associations between risk factors and COVID-19 severity have been consistently reported,^5^ the biological mechanisms linking cardiometabolic risk factors and severe complications of COVID-19 have not been fully elucidated. Thus, measuring circulating proteins could be useful to better understand biological pathways between cardiometabolic factors and complications related to COVID-19. Such biomarkers could be used to stratify patients for the risk of complications associated with COVID-19 and may represent new therapeutic targets for interventions against COVID-19 severity.^6^

Although epidemiologic analyses suffer from bias resulting from both confounding or reverse causation, mendelian randomization (MR) is an established statistical method that uses genetic associations with both a causal factor, also termed the *exposure*, and an outcome to infer causality.^7^ Previous MR studies have identified causal biomarkers involved in COVID-19 severity,^8^ including angiotensin-converting enzyme 2 being considered a key effector.^9^ Yet, to our knowledge, there is no MR study that has investigated which circulating proteins mediate or mitigate the association of cardiometabolic risk factors with COVID-19 severity.

This study aimed to investigate the causal relationships among cardiometabolic risk factors, circulating protein biomarkers, and severe COVID-19. We applied MR analyses to test whether biomarker levels in individuals at high risk of cardiovascular disease are causally associated with COVID-19 hospitalization. To do this, we first identified genetic variants associated with biomarker concentration in individuals with dysglycemia and other cardiovascular risk factors, proceeding to then estimate their association with COVID-19 hospitalization. The findings were then validated in 3 large independent populations. Then, we investigated whether cardiometabolic risk factors could affect the identified biomarker concentrations and, finally, whether the identified biomarkers could augment or mitigate the association of cardiometabolic risk factors with COVID-19 hospitalization. Evidence of causal and/or protective relationships among cardiometabolic risk factors, biomarkers, and adverse prognoses in patients with COVID-19 could guide therapeutic decisions, improve risk stratification for COVID-19 severity, and identify new therapeutic targets.

## Methods

This genetic association study was conducted using data from the Outcome Reduction With Initial Glargine Intervention (ORIGIN) trial (NCT00069784), the Prospective Urban and Rural Epidemiological (PURE) biomarker project, and the COVID-19 Host Genetics Initiative. The study protocol was approved by ethics committees at each study site. All participants provided written informed consent, and a subset of participants also consented to the analysis of stored blood for the biomarker study at baseline. The study followed the Strengthening the Reporting of Genetic Association Studies (STREGA) reporting guideline.

### Discovery Analysis of Biomarker Concentration vs COVID-19 Hospitalization

Using data on hospitalized case participants with confirmed COVID-19, we first conducted a 2-sample MR analysis to identify biomarkers among individuals with dysglycemia and other cardiovascular risk factors causally associated with an adverse COVID-19 prognosis.

#### Population

The ORIGIN trial involved 12 537 participants with T2D, impaired glucose tolerance, or impaired fasting glucose and additional cardiovascular risk factors. Using a factorial design, participants were randomly assigned to insulin glargine vs standard care and to an ω-3 fatty acid supplement vs placebo. To monitor cardiovascular events and other health outcomes, participants were followed for a median of 6.2 (IQR, 5.8-6.8) years. Of the 8494 participants who also consented to the analysis of stored blood for the biomarker study at baseline, 5078 had completed genomic analyses. Ethnicity was self-reported as African, European, or Latin American ancestry. Following genotyping quality control procedures, individuals with unknown race or ethnicity from self-reports and those of African ancestry (due to a small sample size of 243 individuals) were not included in subsequent genetic analyses, resulting in a total sample size of 1931 participants of European Caucasian ancestry and 2216 participants of native Latin American ancestry. The detailed protocol was described previously^10^ (participant characteristics are reported in eTable 1 in Supplement 1).

Data on hospitalization attributed to confirmed COVID-19 were publicly available and were released by the COVID-19 Host Genetics Initiative. These consortium data are from a meta-analysis of 20 cohorts using fixed-effects inverse-variance weighting (IVW), with a sample size of 5773 case participants and 15 497 control participants.^11^ The data come from individuals of various panethnic groups, including African, American, Arab, European, Finnish, Hispanic, and South Asian; although we acknowledge overlap between some of these ethnic and/or cultural groups, these categories were identified by the data set’s contributing studies (participant characteristics are reported in eTable 2 in Supplement 1).^11^ To reduce bias in our analyses due to different population structures during vaccination efforts, we censored the release date from the consortium summary statistics to before the availability of COVID-19 vaccinations (in this case, release 5 from January 18, 2021).^12^

#### Exposure Assessment

Biomarker concentrations were assayed within a multiplex biomarker panel. After completion of the ORIGIN trial, 1 mL of serum from each participant was transported to Myriad RBM Inc to quantify 284 biomarkers (Human Discovery Multi-Analyte Profile 250+ panel on the Luminex 100/200 platform; Luminex) related to metabolic and cardiovascular diseases and high-sensitivity troponin I (Architect Stat assay; Abbott Laboratories). A total of 238 biomarkers from 4147 participants were deemed suitable for analysis^13^ (eTable 3 in Supplement 1). If biomarker concentrations were not normally distributed, they were natural log–transformed to approximate a normal distribution. Values were standardized to a mean (SD) of 0 (1).

#### Genetic Instruments

We selected genetic variants within 300 kb of the biomarker locus (hereinafter, *cis*-protein quantitative trait loci [pQTLs]) and used linear regression analyses to estimate the association between each single-nucleotide variant (SNV) and each of the 238 biomarkers. No genetic instrument could be derived for 3 biomarkers, yielding a total of 235 biomarkers investigated through MR. Models were performed separately for each ethnic group, adjusted for age, sex, and the first 5 principal components (body mass index [BMI; calculated as weight in kilograms divided by height in meters squared], type 2 diabetes [T2D], T2D with BMI adjusted, systolic blood pressure [SBP], and type 1 diabetes [T1D]), and then meta-analyzed across ethnicities using fixed-effects models, as previously described.^10^ Then, *cis*-SNVs associated with biomarker concentrations (*P* < .01) were pruned for linkage disequilibrium at a threshold of *r*^2^ < 0.1.

#### Study Outcomes

The primary outcome for all MR analyses was hospitalization of case participants with confirmed COVID-19, using summary-level data as defined by the COVID-19 Host Genetics Initiative.^11^ Specifically, we used data reporting the phenotype “hospitalized covid vs. not hospitalized covid” (B1_ALL_leave_23andme), release 5, from January 18, 2021.

### Statistical Analysis

#### Two-Sample MR Analysis 

The 2-sample MR analysis assessed the association between biomarker levels from ORIGIN participants and severe COVID-19 using summary association statistics from the COVID-19 Host Genetics Initiative.^11^ To comprehensively screen for associations between biomarkers and COVID-19 severity, we used the IVW method by regressing the genetic effect estimates for COVID-19 hospitalization on the genetic effect estimates for biomarker concentration. To correct for multiple hypothesis testing, *P* < .05/235, *P* = 2.13 × 10^−4^, was considered statistically significant. We then used multiple MR methods to validate any identified associations, specifically using the simple median, weighted median, penalized weighted median, IVW, penalized IVW, robust IVW, and penalized robust IVW. We also performed MR-Egger intercept testing for each univariate MR analysis to test whether the genetic variants had a directional pleiotropic effect (*P* < .05). Analyses were conducted using the MendelianRandomization package^14^ in R, version 3.6.0 (R Project for Statistical Computing).

#### Validation Analysis of Biomarker Concentration vs COVID-19 Hospitalization

The identified biomarkers were then replicated using MR analyses in 3 independent populations in which the pQTLs of biomarkers were available. The first population was from the large prospective PURE study of individuals in 27 low-, middle-, and high-income countries, as described by Narula et al.^9^ A subset of 55 246 PURE participants had blood samples collected at baseline, among which 11 016 participants were selected to constitute a case cohort, with enrichment in cases for incident cardiometabolic events and death.^9^ Participants had undergone genotyping as well as proteomic analyses using the Olink platform (Olink Proteomics), as described by Narula et al.^9^ The other 2 study populations were from Folkersen et al^15^ (n = 3394 participants with at least 3 established cardiovascular disease risk factors but without prior cardiovascular disease at baseline) and Sun et al^16^ (n = 3301 healthy adults of European ancestry), for which biomarker pQTL data were publicly available (participant characteristics are reported in eTable 4 in Supplement 1). Because biomarkers were measured using different technology platforms, including Olink for Narula et al^9^ and Folkersen et al^15^ and SomaScan (SomaLogic) for Sun et al,^16^ MR analyses were performed separately for each study, using methods similar to those used in the initial analysis, including IVW and MR-Egger regression. The odds ratios (ORs) for COVID-19 hospitalization per standardized biomarker levels were then meta-analyzed across the different populations using a fixed-effects meta-analysis in the rmeta package in R.^17^ Fixed-effects analysis was used rather than random-effects analysis, assuming that 1 true effect size applied across all studies considered.

#### Multivariable MR Analysis

To investigate whether the identified biomarkers could augment or mitigate the association of cardiometabolic risk factors with hospitalization related to COVID-19, we estimated the following: (1) whether cardiometabolic risk factors affected the levels of the identified biomarkers, (2) whether cardiometabolic risk factors affected COVID-19 hospitalization, and (3) whether the identified biomarkers promoted or mitigated the association of cardiometabolic factors with COVID-19 hospitalization. Multivariable MR (MVMR) analysis was conducted using the MendelianRandomization package in R, with cardiometabolic risk factors as the exposure, the identified biomarkers as the mediators, and COVID-19 hospitalization as the outcome. The MVMR approach uses multiple genetic variants associated with several measured risk factors to simultaneously estimate the causal relationship between each risk factor on the outcome.^18^ Thus, an MVMR model was fit for the association where cardiometabolic risk factors affected both the biomarker and COVID-19 hospitalization, mediated through the identified biomarker. Genome-wide association study summary-level data of cardiometabolic risk factors were used as inputs for the exposure; specifically, BMI,^19^ T1D,^20^ SBP,^21^ T2D,^22^ and T2D adjusted for BMI^22^ were considered. To design these genetic instruments, we applied a linkage disequilibrium threshold for pruning at *r*^2^ < 0.01 and a threshold of *P* < 5 × 10^−8^ for associations with each cardiometabolic risk factor. Although these populations were mostly of European ancestry, we still used biomarker pQTLs generated in the multiethnic PURE study as inputs for the MVMR. Summary association statistics from the COVID-19 Host Genetics Initiative^11^ were used as inputs for the outcome. Data analyses were performed in July 2022.

## Results

### Biomarker Concentration vs COVID-19 Hospitalization

Among the 235 biomarkers tested using the ORIGIN data, 15 were nominally associated with COVID-19 hospitalization (eTable 5 in Supplement 1). The largest association corresponded to hepatitis A virus cellular receptor 1, also known as kidney injury molecule-1 (KIM-1), in cells other than hepatocytes, such that a higher level of KIM-1 was associated with a decreased risk of COVID-19 hospitalization (OR per SD, 0.86 [95% CI, 0.79-0.93]; *P* = 3.81 × 10^−4^) (Table 1). Analyses using multiple MR methods also confirmed this association (eFigure and eTable 6 in Supplement 1). No directional pleiotropy was detected using the MR-Egger regression model (MR-Egger intercept *P* = .14).

**Table 1.** Mendelian Randomization (MR) Meta-Analysis of the Association of Circulating Kidney Injury Molecule-1 Levels With COVID-19 Hospitalization

Study	OR (95% CI) per 1 − SD biomarker level	*P* value (MR IVW)	Sample size	No. of SNVs
Folkersen et al,^15^ 2017	0.95 (0.90-1.01)	.10	3394	32
Sun et al,^16^ 2018	0.92 (0.71-1.20)	.55	3301	7
Narula et al,^9^ 2020	0.89 (0.82-0.97)	9.0 × 10^−3^	11 016	35
ORIGIN study	0.86 (0.79-0.93)	3.81 × 10^−4^	4390	24
Fixed-effects meta-analysis	0.91 (0.88-0.95)	1.85 × 10^−5^	22 101	NA

Abbreviations: IVW, inverse-variance weighting; NA, not applicable; OR, odds ratio; ORIGIN, Outcome Reduction With Initial Glargine Intervention; SNV, single-nucleotide variant.

We validated the KIM-1 association in 3 independent cohorts (Table 1), and the directionality of the association was consistent across all studies. The meta-analysis of the results, including the discovery sample and the 3 cohorts (N = 22 101), confirmed a protective relationship between KIM-1 levels and COVID-19 hospitalization (OR per SD, 0.91 [95% CI, 0.88-0.95]; *P* = 1.85 × 10^−5^).

### Cardiometabolic Risk Factors vs Biomarker Concentration

Next, we investigated whether cardiometabolic risk factors affected the KIM-1 concentration using 2-sample MR. The results (summarized in Table 2) indicated that of the 5 factors considered, only BMI was associated with KIM-1. Specifically, the KIM-1 concentration increased by 0.17 SD per 1-kg/m^2^ increase in BMI (95% CI, 0.08-0.26; *P* = 1.62 × 10^−4^). There was no evidence of directional pleiotropy (any MR-Egger intercept was significant) and the other cardiovascular risk factors tested, including T1D, T2D, and T2D adjusted for BMI, and SBP, were not associated with KIM-1 levels.

**Table 2.** Association of Genetically Determined Cardiometabolic Risk Factors With Circulating Kidney Injury Molecule-1 (KIM-1) Levels

Risk factor	Change in circulating KIM-1 levels (in SD), mean (95% CI)	*P* value		No. of SNVs
		MR IVW	Intercept (MR Egger)	
BMI	0.17 (0.08 to 0.26)	1.62 × 10^−4^	.15	1116
Type 2 diabetes	−0.002 (−0.063 to 0.059)	.49	.87	39
Type 2 diabetes (BMI adjusted)	−0.003 (−0.065 to 0.058)	.27	.72	54
Systolic blood pressure	0.007 (−0.001 to 0.015)	.09	.71	415
Type 1 diabetes	−0.008 (−0.024 to 0.009)	.35	.34	83

Abbreviations: BMI, body mass index (calculated as weight in kilograms divided by height in meters squared); IVW, inverse-variance weighting; MR, mendelian randomization; SNV, single-nucleotide variant.

### Mediation Analysis of Risk Factors, Biomarkers, and COVID-19 Hospitalization

Finally, we used MR to test whether KIM-1 promoted or mitigated the association of BMI with COVID-19 hospitalization. The univariate MR analyses between risk factors and hospitalization indicated that only BMI was associated with COVID-19 hospitalization, such that risk for hospitalization increased by 33% per 1-kg/m^2^ increase in BMI (OR per 1 kg/m^2^, 1.33 [95% CI, 1.18-1.50]; *P* = 4.45 × 10^−6^), with no evidence of directional pleiotropy (Table 3). We observed that T1D, SBP, T2D, and T2D adjusted for BMI were not associated with hospitalization attributable to COVID-19. The MVMR analysis (including 723 SNVs) indicated that KIM-1 affected the associations between BMI and COVID-19 hospitalization (OR per SD, 1.23 [95% CI, 1.06-1.43]; *P* = 5.65 × 10^−3^). Notably, although BMI levels increased COVID-19 hospitalization, the OR in the KIM-1–mediated pathway was 10 percentage points lower compared with that obtained from the univariate analysis between BMI and COVID-19 hospitalization (Table 3). Thus, BMI was causally associated with increased hospitalization of patients with COVID-19, with KIM-1 decreasing the association of increased BMI with COVID-19 severity by approximately 10% (Figure).

**Table 3.** Association of Genetically Determined Cardiometabolic Risk Factors With COVID-19 Hospitalization

Risk factor	OR for COVID-19 hospitalization (95% CI)	*P* value		No. of SNVs
		MR IVW	Intercept (MR Egger)	
BMI	1.33 (1.18-1.50)	4.45 × 10^−6^	.25	1110
Type 2 diabetes	1.03 (0.95-1.12)	.46	.60	39
Type 2 diabetes (BMI adjusted)	1.06 (0.98-1.15)	.16	.87	54
Systolic blood pressure	1.00 (0.99-1.01)	.45	.05	411
Type 1 diabetes	0.99 (0.97-1.02)	.59	.34	92

Abbreviations: BMI, body mass index (calculated as weight in kilograms divided by height in meters squared); IVW, inverse-variance weighting; MR, mendelian randomization; OR, odds ratio; SNV, single-nucleotide variant.

**Figure.**
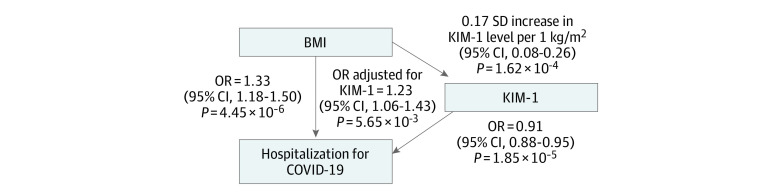
Associations Among Body Mass Index (BMI), Kidney Injury Molecule-1 (KIM-1), and COVID-19 Hospitalization Two-sample mendelian randomization (MR) analysis of associations between BMI and KIM-1, KIM-1 and COVID-19 hospitalization, and BMI and COVID-19 hospitalization. Multivariable MR analysis of the association between BMI and COVID-19 hospitalization, with KIM-1 as a covariate. Odds ratios (ORs) are presented with 95% CIs and *P* values.

## Discussion

In this study, comprehensive screening of 235 circulating protein biomarkers using a 2-sample MR approach identified KIM-1 as a mitigator of COVID-19 severity. The KIM-1 membrane protein is expressed in the kidney, lung, liver, and spleen and plays a role in controlling viral infection and autoimmunity through multiple mechanisms.^23^ Although other biomarkers have been found to better estimate decreased kidney function,^24^ KIM-1 is also an established blood and urine marker of acute kidney injury.^25^ For example, serum KIM-1 levels have been associated with estimated glomerular filtration rate decline.^26^ Although the protective causality between KIM-1 and adverse prognosis is not yet described, the clinical epidemiologic association between KIM-1 and severe COVID-19 was reported previously^27^ and is consistent with our findings. In fact, urinary KIM-1 levels were observed to be elevated in patients with COVID-19, regardless of whether they had acute kidney injury.^28^ Moreover, KIM-1 may act as a receptor for SARS-CoV-2, the virus causing COVID-19, in both lung and kidney epithelia; according to this, the entry and progression of SARS-CoV-2 may be promoted with elevated KIM-1 levels.^29^ With little evidence on the role of KIM-1 in COVID-19 severity, future research is needed to confirm these findings of the potential protective role of KIM-1 in COVID-19 hospitalization.

To our knowledge, this study is the first to report a causal relationship between BMI and KIM-1 levels, which is consistent with a previous epidemiologic study that suggested a positive association between obesity and urinary KIM-1 levels.^30^ Our MR results are also consistent with previous evidence of a causal relationship between BMI and increased COVID-19 susceptibility^31,32,33^ and severity.^31,33^

The MR results observed herein suggest that the association between BMI and risk of severe COVID-19 might be attenuated by KIM-1, although these findings warrant further investigation. One explanation might be that exposure to high BMI chronically increases KIM-1 expression possibly involved in progressive kidney fibrosis and chronic kidney failure, but in acute situations, high KIM-1 expression may have a beneficial phagocytic function of apoptotic bodies that downregulates inflammation and the innate immune response. This early KIM-1–mediated mechanism after ischemic or toxic injury has been well described in acute kidney injury^34^ and has been reported as a natural defense system against acute tubular injury, facilitating tissue repair. Similar adaptive mechanisms might be suspected in the lungs, warranting further study.

Kidney injury molecule-1 is an increasingly studied biomarker for pharmacologic applications in the field of kidney disease.^35,36^ This protein is seen as a potentially druggable therapeutic target (1) in pathways activated by KIM-1 expression to facilitate phagocytic processes^34^ or (2) through the mammalian target of rapamycin pathway.^37^ However, we suspect that the role of KIM-1 is not limited to kidney function, and our results suggest the existence of a biological pathway linking BMI, KIM-1, and COVID-19 severity.

It should be noted that COVID-19 is not the only virus with which KIM-1 been associated. This protein was also determined to be an attachment factor for exosome-packaged hepatitis A virus, working alongside cholesterol transporter Niemann-Pick disease, type C1, in clathrin-mediated endocytosis.^38^ Furthermore, when KIM-1 was associated with immunoglobulin A, hepatitis A virus–receptor interactions were enhanced.^39^ Other evidence also suggests that KIM-1 acts as a receptor or cofactor for *Zaire ebolavirus*^39^ and is a factor for dengue virus entry into target cells.^40^ Promoting cellular entry may not necessarily promote adverse infection. For example, although KIM-1 enhances the cellular internalization of HIV, it also helps inhibit the release of viral particles from the cell, thereby “trapping” the virus within the cell.^41^

Obesity has been associated with unfavorable clinical outcomes for a variety of reasons related to viral infections. For example, a higher BMI has been associated with greater susceptibility to the development of many types of infections, more serious prognoses, and decreased response to vaccinations and antimicrobial drugs.^42^ When considering respiratory viral infections in particular, such as those from H1N1 influenza, obesity is found to be an independent risk factor for disease severity.^43^ Although the biological mechanisms need to be identified, obesity may result in an attenuated antiviral interferon response, thereby worsening viral disease prognosis.^43^ Given this, an association between BMI and COVID-19 hospitalization is expected, and information regarding the potentially attenuating role of KIM-1 may be of clinical interest not only in the acute phase but also during the post-COVID recovery phase.

### Limitations

Although this study benefits from the large sample sizes of the data sets used as exposures, mediators, and outcomes, methodologic limitations remain. The data used in the MR analyses involved mostly European populations, which limits the generalizability of these findings to other ethnic groups.^44^ Furthermore, the data sources we used did not contain individual information on COVID-19 hospitalization; thus, they did not allow for longitudinal assessments or direct assessments of the associations between biomarkers and COVID-19 hospitalization or for analysis stratified by treatment received during hospitalization. Finally, circulating KIM-1 levels might not reflect tissue-specific expression of KIM-1 in virus-targeted organs.

## Conclusions

In this genetic association study of cardiometabolic risk factors, circulating biomarkers (namely, KIM-1), and COVID-19 hospitalization, MR analyses identified a protective relationship between KIM-1 and severe COVID-19, while BMI was shown to upregulate KIM-1 levels. Furthermore, analyses suggested that KIM-1 may attenuate the association between BMI and COVID-19 hospitalization. Although KIM-1 has been detailed with respect to kidney injury and viral responses, this study revealed a pathway linking BMI, KIM-1, and serious COVID-19. However, these results would benefit from further studies.

## Notes

Supplement 1. **eTable 1.** Characteristics of ORIGIN Participants **eTable 2.** Population Descriptive From the COVID-19 Host Genetics Initiative (Release 5) **eTable 3.** List of Biomarkers (ORIGIN) **eTable 4.** Population Descriptive for Replication Analyses **eTable 5.** Mendelian Randomization (MR) Results of Biomarkers Nominally Associated With COVID-19 Hospitalization **eTable 6.** Associations Between Circulating Kidney Injury Molecule-1 (KIM-1) Levels and COVID-19 Hospitalization, Using Different Mendelian Randomization (MR) Methods **eFigure.** Graphical Representation of the Association Between Circulating Kidney Injury Molecule-1 (KIM-1) Levels (Exposure) and COVID-19 Hospitalization (Outcome), Using Different Mendelian Randomization (MR) Methods

Supplement 2.
Data Sharing Statement
