# Supplementary material for: Biomarkers Associated With Severe COVID-19 Among Populations With High Cardiometabolic Risk: A 2-Sample Mendelian Randomization Study
Source: JAMA Netw Open. 2023 Jul 27;6(7):e2325914. doi: 10.1001/jamanetworkopen.2023.25914 (PMC10375306; doi:10.1001/jamanetworkopen.2023.25914)
Supplement: Supplement 1. — eTable 1. Characteristics of ORIGIN Participants eTable 2. Population Descriptive From the COVID-19 Host Genetics Initiative (Release 5) eTable 3. List of Biomarkers (ORIGIN) eTable 4. Population Descriptive for Replication Analyses eTable 5. Mendelian Randomization (MR) Results of Biomarkers Nominally Associated With COVID-19 Hospitalization eTable 6. Associations Between Circulating Kidney Injury Molecule-1 (KIM-1) Levels and COVID-19 Hospitalization, Using Different Mendelian Randomization (MR) Methods eFigure. Graphical Representation of the Association Between Circulating Kidney Injury Molecule-1 (KIM-1) Levels (Exposure) and COVID-19 Hospitalization (Outcome), Using Different Mendelian Randomization (MR) Methods [file jamanetwopen-e2325914-s001.pdf]

Supplemental Online Content

Sood T, Perrot N, Chong M, et al. Biomarkers associated with severe COVID-19 among populations with high cardiometabolic risk: a 2-sample mendelian randomization study. *JAMA Netw Open*. 2023;6(7):e2325914. doi:10.1001/jamanetworkopen.2023.25914

eTable 1. Characteristics of ORIGIN Participants

eTable 2. Population Descriptive From the COVID-19 Host Genetics Initiative (Release 5)

eTable 3. List of Biomarkers (ORIGIN)

eTable 4. Population Descriptive for Replication Analyses

eTable 5. Mendelian Randomization (MR) Results of Biomarkers Nominally Associated With COVID-19 Hospitalization

eTable 6. Associations Between Circulating Kidney Injury Molecule-1 (KIM-1) Levels and COVID-19 Hospitalization, Using Different Mendelian Randomization (MR) Methods

eFigure. Graphical Representation of the Association Between Circulating Kidney Injury Molecule-1 (KIM-1) Levels (Exposure) and COVID-19 Hospitalization (Outcome), Using Different Mendelian Randomization (MR) Methods

This supplemental material has been provided by the authors to give readers additional information about their work.

© 2023 Sood T et al. *JAMA Network Open*.

eTable 1. Characteristics of ORIGIN Participants

Variables	Proteomic study participants (n = 8,197)	Genetic study participants (n = 4,147)
Age (years)	63.72 (7.94)	63.45 (7.98)
Sex (% male)	66.11	64.14
Ancestry - Europeans (%)	55.41	46.56
- Latin Americans (%)	34.28	53.44
- South Asian (%)	5.49	-
- Black (%)	4.36	-
- South East Asian (%)	0.46	-
Smoking (%, ever)	60.10	60.16
Body mass index (kg/m^2)	30.04 (5.27)	30.45 (5.33)
Prior type 2 diabetes (%)	81.66	87.56
Fasting plasma glucose (mmol/L)	7.33 (2.02)	7.58 (2.17)
HbA_1c (%)	6.5 (0.95)	6.6 (0.98)
(mmol/mol)	48.0 (10.4)	49.0 (10.7)
Prior hypertension (%, yes)	78.91	82.9
LDL (mmol/L)	2.89 (1.03)	3.07 (1.05)
HDL (mmol/L)	1.18 (0.32)	1.17 (0.32)
Triglycerides (mmol/L)	1.89 (1.24)	1.93 (1.17)
Prior cardio-vascular event (%, yes)	59.57	53.29

Data are presented as the mean (SD) unless stated otherwise.

© 2023 Sood T et al. *JAMA Network Open*.

eTable 2. Population Descriptive From the COVID-19 Host Genetics Initiative (Release 5)

Phenotype	Hospitalized covid vs. not hospitalized covid		
	Nb cases	Nb controls	Demographics
BelCovid_EUR	361	122	Cases 67 years<br>Controls 56 years
BoSCO_EUR	212	512	Cases 48.40 years
EstBB_EUR	60	512	Cases 50.9 years<br>Controls 48.9 years
FHoGID_EUR	362	259	Cases 65.43 years<br>Controls 51.07 years
FinnGen_FIN	106	520	Cases 51.8 years<br>Controls 58.6 years
GENCOVID_EUR	892	249	Cases 63.29 years<br>Controls 48.15 years
GNH_SAS	115	1264	Cases & Controls 39.5 years
UCLA_AMR	95	74	Cases & Controls > 50 years
UCLA_EUR	80	123	Cases & Controls > 50 years
UKBB_AFR	71	119	Cases 69.52 years<br>Controls 67.92 years
UKBB_EUR	1670	4610	Cases 69.52 years<br>Controls 67.92 years
UKBB_SAS	71	230	Cases 69.52 years<br>Controls 67.92 years
SPGRX_EUR	311	51	Cases 69.32 years<br>Controls 47.92 years
DECODE_EUR	89	1808	Cases & Controls 42 years
PMBB_AFR	66	100	Cases 49.5 years<br>Controls 58.2 years
QGP_ARAB	60	640	Cases & Controls 40.05 years
MVP_AFR	349	862	Cases 65.6 years<br>Controls 66.5 years
MVP_EUR	436	1083	Cases 65.6 years<br>Controls 66.5 years
MVP_HIS	117	392	Cases 65.6 years<br>Controls 66.5 years
Ancestry_EUR	250	1967	Cases 54.7 years<br>Controls 52.1 years
Total	5,773	15497	

Downloaded from :
https://storage.googleapis.com/covid19-hg-public/20201215/results/20210107/COVID19_HGI_B1_ALL_leave_23andme_20210107.b37.txt.gz.tbi

© 2023 Sood T et al. *JAMA Network Open*.

eTable 3. List of Biomarkers (ORIGIN)

	**Biomarker**	**Gene**	**Inter-Run CV at Intermediate Concentrations**
**1**	6Ckine	*CCL21*	13%
**2**	Adiponectin	*ADIPOQ*	4%
**3**	Adrenomedullin	*ADM*	7%
**4**	Agouti-Related Protein	*AGRP*	7%
**5**	Aldose Reductase	*AKR1B1*	11%
**6**	Alpha-1-acid glycoprotein 1	*ORM1*	15%
**7**	Alpha-1-Antichymotrypsin	*SERPINA3*	6%
**8**	Alpha-1-Antitrypsin	*SERPINA1*	14%
**9**	Alpha-1-Microglobulin	*AMBP*	5%
**10**	Alpha-2-Macroglobulin	*A2M*	6%
**11**	Angiogenin	*ANG*	10%
**12**	Angiopoietin-2	*ANGPT2*	6%
**13**	Angiopoietin-related protein 3	*ANGPTL3*	8%
**14**	Angiotensin-Converting Enzyme	*ACE*	12%
**15**	Angiotensinogen	*AGT*	6%
**16**	Antithrombin-III	*SERPINC1*	6%
**17**	Apolipoprotein A-I	*APOA1*	10%
**18**	Apolipoprotein A-II	*APOA2*	8%
**19**	Apolipoprotein A-IV	*APOA4*	9%
**20**	Apolipoprotein B	*APOB*	9%
**21**	Apolipoprotein C-I	*APOC1*	9%
**22**	Apolipoprotein C-III	*APOC3*	20%
**23**	Apolipoprotein D	*APOD*	18%
**24**	Apolipoprotein E	*APOE*	20%
**25**	Apolipoprotein H	*APOH*	12%
**26**	Apolipoprotein(a)	*LPA*	16%
**27**	AXL Receptor Tyrosine Kinase	*AXL*	10%
**28**	B cell-activating factor	*TNFSF13B*	9%
**29**	B Lymphocyte Chemoattractant	*CXCL13*	10%
**30**	Beta Amyloid 1-40	*APP*	13%
**31**	Beta-2-Microglobulin	*B2M*	13%
**32**	Brain-Derived Neurotrophic Factor	*BDNF*	5%
**33**	C-Peptide	*INS*	4%

© 2023 Sood T et al. *JAMA Network Open*.

**34**	C-Reactive Protein	*CRP*	13%
**35**	Cathepsin D	*CTSD*	9%
**36**	CD 40 antigen	*CD40*	8%
**37**	CD163	*CD163*	11%
**38**	CD40 Ligand	*CD40LG*	9%
**39**	CD5 Antigen-like	*CD5L*	14%
**40**	Cellular Fibronectin	*FN1*	17%
**41**	Chemerin	*RARRES2*	4%
**42**	Chemokine CC-4	*CCR4*	7%
**43**	Chromogranin-A	*CHGA*	7%
**44**	Clusterin	*CLU*	10%
**45**	Collagen IV	*COL4A1,COL4A2,COL4A3,COL4A4,COL4A5,COL4A6*	14%
**46**	Complement C3	*C3*	10%
**47**	Complement Factor H Related Protein 1	*CFHR1*	9%
**48**	Cortisol	*NA*	8%
**49**	Creatine Kinase-MB	*CKM,CKB*	12%
**50**	Cystatin-C	*CST3*	8%
**51**	E-Selectin	*SELE*	5%
**52**	EN-RAGE	*S100A12*	4%
**53**	Endoglin	*ENG*	8%
**54**	Endostatin	*COL18A1*	10%
**55**	Eotaxin-1	*CCL11*	11%
**56**	Eotaxin-2	*CCL24*	9%
**57**	Eotaxin-3	*CCL26*	16%
**58**	Epithelial-Derived Neutrophil-Activating Protein 78	*CXCL5*	12%
**59**	Erythropoietin	*EPO*	10%
**60**	Ezrin	*EZR*	10%
**61**	Factor VII	*F7*	4%
**62**	Fas Ligand	*FASLG*	8%
**63**	FASLG Receptor	*TNFRSF6B*	2%
**64**	Fatty Acid-Binding Protein adipocyte	*FABP4*	6%
**65**	Fatty Acid-Binding Protein liver	*FABP1*	12%
**66**	Ferritin	*FTL,FTH1*	6%
**67**	Fetuin-A	*AHSG*	17%
**68**	Fibroblast Growth Factor 21	*FGF21*	12%

© 2023 Sood T et al. *JAMA Network Open*.

**69**	Fibroblast growth factor 23	*FGF23*	9%
**70**	Fibulin-1C	*FBLN1*	9%
**71**	Ficolin-3	*FCN3*	7%
**72**	Follicle-Stimulating Hormone	*FSHB,CGA*	16%
**73**	Galectin-3	*LGALS3*	8%
**74**	Gastric inhibitory polypeptide	*GIP*	12%
**75**	Gelsolin	*GSN*	12%
**76**	Glucagon-like Peptide 1 total	*GCG*	7%
**77**	Glucose-6-phosphate Isomerase	*GPI*	7%
**78**	Glutathione S-Transferase alpha	*GSTA1,GSTA2,GSTA3,GSTA4,GSTA5*	11%
**79**	Glycogen phosphorylase isoenzyme BB	*PYGB*	6%
**80**	Granulocyte Colony-Stimulating Factor	*CSF3*	6%
**81**	Growth differentiation factor 15	*GDF15*	10%
**82**	Growth Hormone	*GH1,GH2*	9%
**83**	Growth-Regulated alpha protein	*CXCL1*	5%
**84**	Haptoglobin	*HP*	8%
**85**	Heat-Shock protein 70	*HSPA1A,HSPA1B,HSPA1L,HSPA2,HSPA4,HSPA4L,HSPA5,HSPA6,HSPA8,HSPA9,HSPA12A,HSPA12B,HSPA13,HSPA14*	8%
**86**	Hemopexin	*HPX*	9%
**87**	Hepatocyte Growth Factor	*HGF*	18%
**88**	Hepatocyte Growth Factor receptor	*MET*	13%
**89**	Hepsin	*HPN*	4%
**90**	Human Epidermal Growth Factor Receptor 2	*ERBB2*	2%
**91**	Immunoglobulin A	*IGH*	11%
**92**	Immunoglobulin E	*IGH*	4%
**93**	Immunoglobulin M	*IGH*	19%
**94**	Insulin	*INS*	7%
**95**	Insulin-like Growth Factor Binding Protein 4	*IGFBP4*	6%

© 2023 Sood T et al. *JAMA Network Open*.

**96**	Insulin-like Growth Factor Binding Protein 5	*IGFBP5*	8%
**97**	Insulin-like Growth Factor Binding Protein 6	*IGFBP6*	17%
**98**	Insulin-like Growth Factor I	*IGF1*	8%
**99**	Insulin-like Growth Factor-Binding Protein 1	*IGFBP1*	8%
**100**	Insulin-like Growth Factor-Binding Protein 2	*IGFBP2*	7%
**101**	Insulin-like Growth Factor-Binding Protein 3	*IGFBP3*	9%
**102**	Intercellular Adhesion Molecule 1	*ICAM1*	7%
**103**	Interferon gamma	*IFNG*	11%
**104**	Interferon gamma Induced Protein 10	*CXCL10*	8%
**105**	Interferon-inducible T-cell alpha chemoattractant	*CXCL11*	16%
**106**	Interleukin-1 beta	*IL1B*	10%
**107**	Interleukin-1 receptor antagonist	*IL1RN*	6%
**108**	Interleukin-10	*IL10*	8%
**109**	Interleukin-12 Subunit p40	*IL12B*	7%
**110**	Interleukin-16	*IL16*	5%
**111**	Interleukin-17	*IL17A*	6%
**112**	Interleukin-18	*IL18*	9%
**113**	Interleukin-2	*IL2*	8%
**114**	Interleukin-2 receptor alpha	*IL2RA*	2%
**115**	Interleukin-23	*IL23A,IL12B*	9%
**116**	Interleukin-6	*IL6*	6%
**117**	Interleukin-6 receptor	*IL6R*	6%
**118**	Interleukin-6 receptor subunit beta	*IL6ST*	9%
**119**	Interleukin-7	*IL7*	6%
**120**	Interleukin-8	*CXCL8*	8%
**121**	Kallikrein 5	*KLK5*	14%
**122**	Kidney Injury Molecule-1	*HAVCR1*	9%
**123**	Lactoferrin	*LTF*	9%
**124**	Lactoylglutathione lyase	*GLO1*	6%
**125**	Latency-Associated Peptide of Transforming Growth Factor beta 1	*LTBP1*	7%
**126**	Lectin-Like Oxidized LDL Receptor 1	*OLR1*	4%

© 2023 Sood T et al. *JAMA Network Open*.

**127**	Leptin	*LEP*	6%
**128**	Leptin Receptor	*LEPR*	11%
**129**	Leucine-rich alpha-2-glycoprotein	*LRG1*	4%
**130**	Luteinizing Hormone	*LHB,CGA*	6%
**131**	Macrophage Colony-Stimulating Factor 1	*CSF1*	5%
**132**	Macrophage inflammatory protein 3 beta	*CCL19*	10%
**133**	Macrophage Inflammatory Protein-1 alpha	*CCL3*	7%
**134**	Macrophage Inflammatory Protein-1 beta	*CCL4*	6%
**135**	Macrophage Inflammatory Protein-3 alpha	*CCL20*	4%
**136**	Macrophage Migration Inhibitory Factor	*MIF*	6%
**137**	Macrophage-Derived Chemokine	*CCL22*	9%
**138**	Macrophage-Stimulating Protein	*MST1*	6%
**139**	Matrix Metalloproteinase-1	*MMP1*	12%
**140**	Matrix Metalloproteinase-10	*MMP10*	11%
**141**	Matrix Metalloproteinase-3	*MMP3*	9%
**142**	Matrix Metalloproteinase-7	*MMP7*	12%
**143**	Matrix Metalloproteinase-9	*MMP9*	6%
**144**	Matrix Metalloproteinase-9 total	*MMP9*	14%
**145**	Mesothelin	*MSLN*	10%
**146**	Methylglyoxal	*NA*	9%
**147**	MHC class I chain-related protein A	*MICA*	5%
**148**	Monocyte Chemotactic Protein 1	*CCL2*	5%
**149**	Monocyte Chemotactic Protein 2	*CCL8*	6%
**150**	Monocyte Chemotactic Protein 3	*CCL7*	7%
**151**	Monocyte Chemotactic Protein 4	*CCL13*	7%
**152**	Monokine Induced by Gamma Interferon	*CXCL9*	10%
**153**	Myeloid Progenitor Inhibitory Factor 1	*CCL23*	4%

© 2023 Sood T et al. *JAMA Network Open*.

**154**	Myeloperoxidase	*MPO*	16%
**155**	Myoglobin	*MB*	6%
**156**	N-terminal prohormone of brain natriuretic peptide	*NPPB*	5%
**157**	Neuronal Cell Adhesion Molecule	*NRCAM*	5%
**158**	Neuropilin-1	*NRP1*	11%
**159**	Neutrophil Activating Peptide 2	*PPBP*	7%
**160**	Neutrophil Gelatinase-Associated Lipocalin	*LCN2*	12%
**161**	Omentin	*ITLN1*	11%
**162**	Osteocalcin	*BGLAP*	9%
**163**	Osteopontin	*SPP1*	9%
**164**	Osteoprotegerin	*TNFRSF11B*	9%
**165**	P-Selectin	*SELP*	6%
**166**	Pancreatic Polypeptide	*PPY*	13%
**167**	Paraoxanase-1	*PON1*	16%
**168**	Pentraxin-3	*PTX3*	12%
**169**	Pepsinogen I	*NA*	6%
**170**	Peptide YY	*PYY*	7%
**171**	Periostin	*POSTN*	11%
**172**	Peroxiredoxin-4	*PRDX4*	8%
**173**	Phosphoserine Aminotransferase	*PSAT1*	7%
**174**	Pigment Epithelium Derived Factor	*SERPINF1*	7%
**175**	Plasminogen Activator Inhibitor 1	*SERPINE1*	8%
**176**	Platelet-Derived Growth Factor BB	*PDGFB*	6%
**177**	Progesterone	*NA*	8%
**178**	Progranulin	*GRN*	6%
**179**	Proinsulin Intact	*INS*	5%
**180**	Proinsulin Total	*INS*	6%
**181**	Prolactin	*PRL*	15%
**182**	Prostasin	*PRSS8*	6%
**183**	Prostatic Acid Phosphatase	*ACPP*	11%
**184**	Protein S100-A4	*S100A4*	7%
**185**	Protein S100-A6	*S100A6*	8%
**186**	Pulmonary and Activation-Regulated Chemokine	*CCL18*	10%

© 2023 Sood T et al. *JAMA Network Open*.

**187**	Receptor for advanced glycosylation end products	*AGER*	5%
**188**	Receptor tyrosine-protein kinase erbB-3	*ERBB3*	10%
**189**	Resistin	*RETN*	8%
**190**	Retinol-binding protein 4	*RBP4*	13%
**191**	Secreted frizzled-related protein 4	*SFRP4*	12%
**192**	Selenoprotein P	*SEPP1*	6%
**193**	Serotransferrin	*TF*	8%
**194**	Serum Amyloid A Protein	*SAA1,SAA2,SAA4,SAA3P*	11%
**195**	Serum Amyloid P-Component	*APCS*	10%
**196**	Serum Glutamic Oxaloacetic Transaminase	*GOT1,GOT2*	12%
**197**	Sex Hormone-Binding Globulin	*SHBG*	14%
**198**	Sortilin	*SORT1*	5%
**199**	ST2	*IL1RL1*	11%
**200**	Stem Cell Factor	*KITLG*	6%
**201**	Stromal cell-derived factor-1	*CXCL12*	10%
**202**	Superoxide Dismutase 1 soluble	*SOD1*	7%
**203**	T Lymphocyte-Secreted Protein I-309	*CCL1*	11%
**204**	T-Cell-Specific Protein RANTES	*CCL5*	16%
**205**	Tamm-Horsfall Urinary Glycoprotein	*UMOD*	17%
**206**	Tenascin-C	*TNC*	4%
**207**	Testosterone Total	*NA*	7%
**208**	Tetranectin	*CLEC3B*	15%
**209**	Thrombin-activable fibrinolysis inhibitor	*CPB2*	5%
**210**	Thrombomodulin	*THBD*	7%
**211**	Thrombospondin-1	*THBS1*	14%
**212**	Thyroid-Stimulating Hormone	*TSHB,CGA*	8%
**213**	Thyroxine-Binding Globulin	*SERPINA7*	15%
**214**	Tissue Inhibitor of Metalloproteinases 1	*TIMP1*	7%

© 2023 Sood T et al. *JAMA Network Open*.

**215**	Tissue type Plasminogen activator	*PLAT*	5%
**216**	TNF-Related Apoptosis-Inducing Ligand Receptor 3	*TNFRSF10C*	7%
**217**	Transthyretin	*TTR*	8%
**218**	Trefoil Factor 3	*TFF3*	7%
**219**	Troponin		4%
**220**	Tumor Necrosis Factor alpha	*TNF*	5%
**221**	Tumor necrosis factor receptor 2	*TNFRSF1B*	7%
**222**	Tumor Necrosis Factor Receptor I	*TNFRSF1A*	8%
**223**	Tyrosine kinase with Ig and EGF homology domains 2	*TIE1*	7%
**224**	Urokinase-type Plasminogen Activator	*PLAU*	9%
**225**	Urokinase-type plasminogen activator receptor	*PLAUR*	8%
**226**	Vascular Cell Adhesion Molecule-1	*VCAM1*	8%
**227**	Vascular Endothelial Growth Factor	*VEGFA*	7%
**228**	Vascular Endothelial Growth Factor C	*VEGFC*	10%
**229**	Vascular endothelial growth factor D	*FIGF*	7%
**230**	Vascular Endothelial Growth Factor Receptor 2	*FLT1*	6%
**231**	Vascular endothelial growth factor receptor 3	*FLT4*	7%
**232**	Visceral adipose tissue derived serpin A12	*SERPINA12*	7%
**233**	Visfatin	*NAMPT*	15%
**234**	Vitamin D-Binding Protein	*GC*	11%
**235**	Vitamin K-Dependent Protein S	*PROS1*	9%
**236**	Vitronectin	*VTN*	18%
**237**	von Willebrand Factor	*VWF*	12%
**238**	YKL-40	*CHI3L1*	8%

The respective CVs are from the customized validation report provided by Myriad RBM Inc. for the biomarkers assayed for this study; the company's White Paper on Quality systems was accessed at https://myriadrbm.com/scientific-media/quality-control-systems-white-paper/.

© 2023 Sood T et al. *JAMA Network Open*.

eTable 4. Population Descriptive for Replication Analyses

Data sources	Sun et al	Folkersen et al	Narula et al
Study population name	INTERVAL	IMPROVE	PURE
Sample size	3,301	3,394	11,016
Population characteristics	healthy adult participants	participants with at least 3 established CVD risk factors, but without prior CVD at baseline	case-cohort with cases as follows: death (n=1985), CVD death (n=561), MI (n=882), stroke (n=663), HF (n=264), diabetes (1715)
Sex (Nb of men)	1,686 men	3,711 men	5,803 men
Age (years)	mean 43.72 (14.26)	median 64.5 (IQR 59.6 to 67.2)	mean 53.94 (9.27)
Ethnicity	European ancestry	European ancestry	Multiple ancestries
Assay to measure KIM-1	Somalogic	Olink	Olink

© 2023 Sood T et al. *JAMA Network Open*.

eTable 5. Mendelian Randomization (MR) Results of Biomarkers Nominally Associated With COVID-19 Hospitalization

Biomarker	OR (95% CI) per 1 SD biomarker level	P-value (MR IVW)	Intercept P-value (MR-Egger)	Number of SNPs
Kidney injury molecule 1 (KIM-1)	0.86 (0.79, 0.93)	3.81×10^{-4}	1.38×10^{-1}	24
Prostatic acid phosphatase (ACPP)	1.26 (1.11, 1.44)	5.68×10^{-4}	1.83×10^{-1}	18
MHC class I chain-related protein A (MICA)	0.91 (0.86, 0.96)	8.85×10^{-4}	1.60×10^{-1}	34
Apolipoprotein E (APOE)	0.89 (0.83, 0.96)	1.88×10^{-3}	8.42×10^{-1}	25
Cathepsin D (CTSD)	1.13 (1.04, 1.23)	4.40×10^{-3}	3.74×10^{-1}	26
Lactoferrin (LTF)	1.11 (1.03, 1.20)	6.20×10^{-3}	4.80×10^{-1}	27
Thrombospondin-1 (THBS1)	1.21 (1.04, 1.40)	1.08×10^{-2}	5.68×10^{-1}	11
Interleukin-2 receptor alpha (IL2RA)	0.90 (0.83, 0.98)	1.22×10^{-2}	2.35×10^{-1}	23
Myeloperoxidase (MPO)	1.91 (1.10, 3.34)	2.25×10^{-2}	NC	2
Interleukin-6 receptor subunit beta (IL6ST)	0.88 (0.78, 0.99)	2.76×10^{-2}	7.18×10^{-1}	17
Angiotensinogen (AGT)	1.09 (1.01, 1.17)	2.88×10^{-2}	6.16×10^{-1}	27
Tenascin-C (TNC)	0.89 (0.80, 0.99)	3.50×10^{-2}	4.93×10^{-1}	23
Resistin (RETN)	0.80 (0.64, 0.99)	3.78×10^{-2}	7.94×10^{-1}	13
Glutathione S-transferase alpha (GSTA4)	0.44 (0.20, 0.97)	4.18×10^{-2}	NC	1
Platelet-derived growth factor BB (PDGFB)	0.63 (0.41, 0.99)	4.57×10^{-2}	6.45×10^{-1}	3

Legend: MR analysis included independent genetic variants associated with type 2 diabetes in the ORIGIN trial. A two-sample MR analysis applied to the COVID-19 Host Genetics Initiative was used to test for causal associations between biomarkers (in patients with dysglycemia and other CVD risk factors) and hospitalization for COVID-19. “NC” = not calculable.

© 2023 Sood T et al. *JAMA Network Open*.

eTable 6. Associations Between Circulating Kidney Injury Molecule-1 (KIM-1) Levels and COVID-19 Hospitalization, Using Different Mendelian Randomization (MR) Methods

Method	OR (95% CI) per 1 SD biomarker level	P-value
Simple median	0.88 (0.77, 1.00)	5.80×10^{-2}
Weighted median	0.87 (0.77, 0.99)	2.78×10^{-2}
Penalized weighted median	0.87 (0.77. 0.99)	2.78×10^{-2}
IVW	0.86 (0.79, 0.93)	3.81×10^{-4}
Penalized IVW	0.86 (0.79, 0.93)	3.81×10^{-4}
Robust IVW	0.86 (0.81, 0.92)	5.92×10^{-6}
Penalized robust IVW	0.86 (0.81, 0.92)	5.92×10^{-6}
MR-Egger	0.77 (0.65, 0.91)	2.29×10^{-3}
(intercept)	1.03 (0.99, 1.08)	1.38×10^{-1}
Penalized MR-Egger	0.77 (0.65, 0.91)	2.29×10^{-3}
(intercept)	1.03 (0.99, 1.08)	1.38×10^{-1}
Robust MR-Egger	0.78 (0.67, 0.91)	1.20×10^{-3}
(intercept)	1.03 (0.99, 1.07)	9.66×10^{-2}
Penalized robust MR-Egger	0.78 (0.67, 0.91)	1.20×10^{-3}
(intercept)	1.03 (0.99, 1.07)	9.66×10^{-2}

© 2023 Sood T et al. *JAMA Network Open*.

eFigure. Graphical Representation of the Association Between Circulating Kidney Injury Molecule-1 (KIM-1) Levels (Exposure) and COVID-19 Hospitalization (Outcome), Using Different Mendelian Randomization (MR) Methods

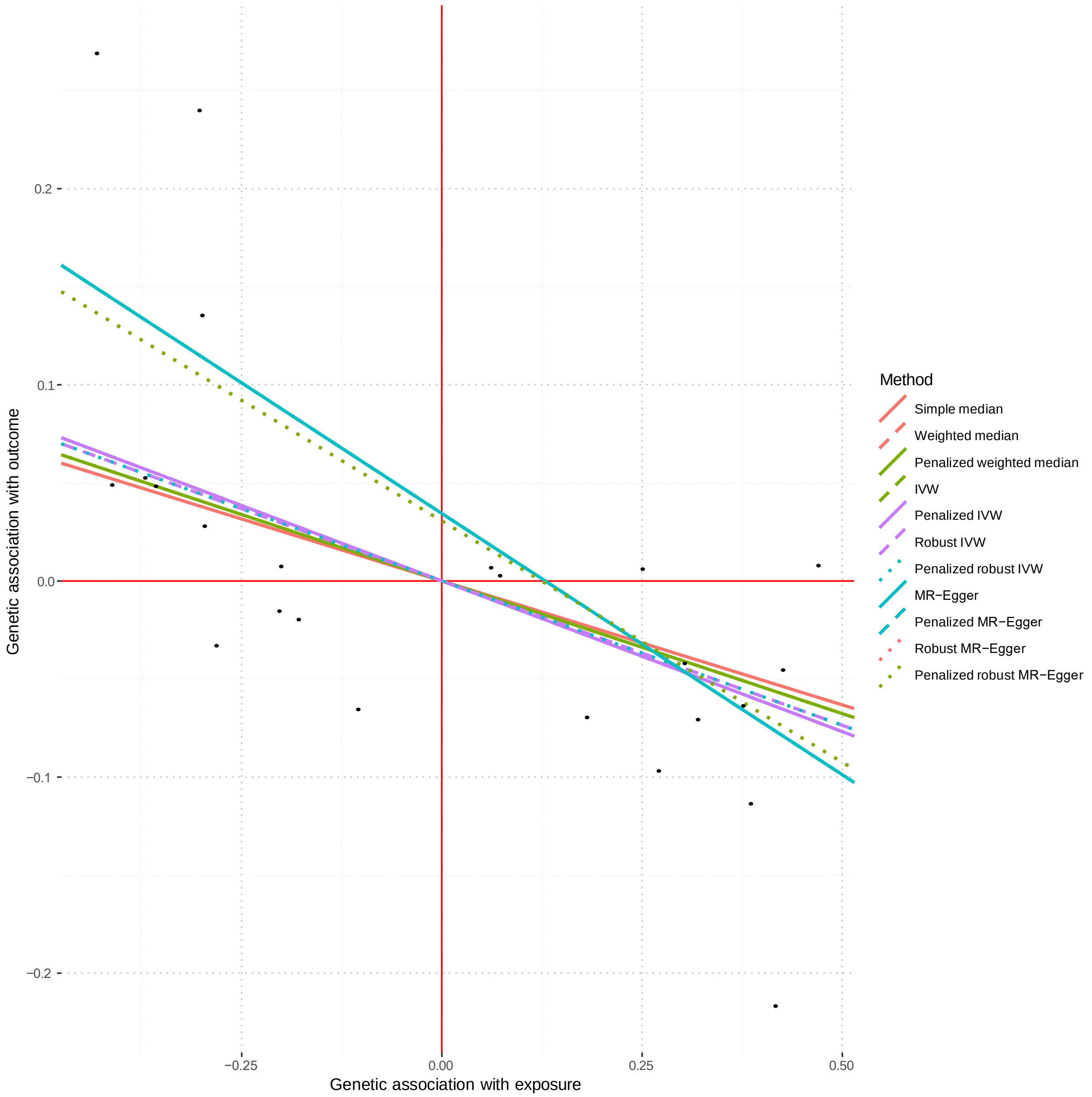

© 2023 Sood T et al. *JAMA Network Open*.
