# Supplementary material for: Biomarkers Associated With Severe COVID-19 Among Populations With High Cardiometabolic Risk: A 2-Sample Mendelian Randomization Study
Source: JAMA Netw Open. 2023 Jul 27;6(7):e2325914. doi: 10.1001/jamanetworkopen.2023.25914 (PMC10375306; doi:10.1001/jamanetworkopen.2023.25914)
Supplement: Supplement 2. — Data Sharing Statement [file jamanetwopen-e2325914-s002.pdf]

Data Sharing Statement

Sood. Biomarkers Associated With Severe COVID-19 Among Populations With High Cardiometabolic Risk. *JAMA Netw Open*. Published July 27, 2023.
doi:10.1001/jamanetworkopen.2023.25914

Data
Data available: No

Additional Information
Explanation for why data not available: only genetic summary statistics may be shared upon request
